# High Expression of G6PD Increases Doxorubicin Resistance in Triple Negative Breast Cancer Cells by Maintaining GSH Level

**DOI:** 10.7150/ijbs.65555

**Published:** 2022-01-01

**Authors:** Man Luo, Afu Fu, Renfei Wu, Na Wei, Kai Song, Sierin Lim, Kathy Qian Luo

**Affiliations:** 1 Interdisciplinary Graduate School, Nanyang Technological University, Singapore.; 2 School of Chemical and Biomedical Engineering, Nanyang Technological University, Singapore.; 3 Faculty of Health Sciences, University of Macau, Macao SAR, China.; 4 Department of Bioengineering, University of California, Los Angeles, CA, USA.; 5 Ministry of Education-Frontiers Science Center for Precision Oncology, University of Macau, Taipa, Macao SAR, China.

**Keywords:** Triple negative breast cancer, Metastasis, Doxorubicin, Drug resistance, Oxidative stress, G6PD

## Abstract

Resistance to doxorubicin (DOX) remains a big challenge to breast cancer treatment especially for triple negative breast cancer (TNBC). Our previous study revealed that the antioxidant system plays an important role in conferring metastasis derived DOX resistance. In this study, we used two-dimensional difference gel electrophoresis (2D-DIGE) proteomics to compare the expression profiles of two generations of TNBC cell lines which have increased metastatic ability in nude mice and exhibited resistance to DOX. Through careful analyses, one antioxidant protein: glucose-6-phosphate dehydrogenase (G6PD) was identified with 3.2-fold higher level in metastatic/DOX-resistant 231-M1 than its parental 231-C3 cells. Analyses of clinical data showed that TNBC patients with higher G6PD levels exhibited lower overall survival than patients with lower G6PD level. Reducing G6PD expression by siRNA or inhibiting its activity with dehydroepiandrosterone (DHEA) significantly increased DOX's cytotoxicity in both cell lines. Importantly, inhibiting G6PD's activity with DHEA dramatically increased the apoptotic rate of 1.25 µM DOX from 2% to 54%. Our results suggest that high level of G6PD can help TNBC to resist DOX-induced oxidative stress. Thus, inhibiting G6PD shall be a good strategy to treat DOX-resistant TNBC.

## Introduction

Treatment for triple negative breast cancer (TNBC) remains a big challenge in clinical practice. Because TNBC cells express neither estrogen receptor (ER) nor progesterone receptor (PR) and nor overexpress human epidermal growth factor receptor 2 (HER2), they respond poorly to either hormone or HER2 targeted therapy 1. Chemotherapy is the backbone of systemic treatment for TNBC, among which anthracyclines are important agents and represent the current standard treatment option 2,3. Several anthracycline antibiotics are available now, among which doxorubicin (DOX) is currently considered as one of the most effective drugs to treat TNBC. However, both early relapse of TNBC leading to devastating consequences on cancer patients and premature development of drug resistance resulting in unsuccessful treatment outcome were observed by clinicians 4,5. Compared with other types of breast cancer, TNBC has a higher recurrence rate within 3 years of diagnosis and an early visceral metastasis 4,6. The breast cancer cells can spread to other sites in the body, including lung, bone, liver, pleura, adrenal glands, among which around 65% of distant metastasis involves lung 7. The metastatic breast cancer remains almost an incurable disease with a five-year survival probability of only around 25%. More than 90% of metastatic breast cancer patients suffered from treatment failure due to chemotherapy resistance 3.

DOX has been used to treat a wide range of cancers for more than four decades. DOX mainly uses two mechanisms to kill cancer cells: first by inhibiting the function of topoisomerase II, which interferes with DNA synthesis; and second by producing superoxide that generates intracellular oxidative stress 8-11. The process of DOX to generate superoxide is as follows: the quinone moiety of DOX first loses one electron to form the semiquinone radical, which causes auto-oxidation in the existing molecular oxygen, resulting in superoxide radical formation 12. When the oxidative stress overwhelms the antioxidant ability of cancer cells, cell apoptosis is triggered 13. In our recent study, we found that superoxide generation is one of the mechanisms of DOX-induced cancer cell apoptosis and an antioxidant protein, manganese superoxide dismutase (MnSOD) is involved in the DOX resistance 14.

In the current study, glucose-6-phosphate dehydrogenase (G6PD) was identified by 2D-DIGE proteomics after comparing the protein profiles between metastatic and original TNBC cells. G6PD was subsequently validated to be an important antioxidant regulator that could mediate metastasis derived DOX resistance. Cancer cells are known to develop increased antioxidant ability to avoid oxidative damages caused by either endogenous or exogenous reactive oxygen species (ROS), such as oxidative stress induced by anti-cancer drugs 15. The production of glutathione (GSH), one of the most abundant intracellular antioxidants, has been shown to be up-regulated in cancer cells 16. The oxidative branch of pentose phosphate pathway (PPP) is a major source of nicotinamide adenine dinucleotide phosphate (NADPH), which is essential for generating intracellular GSH 17. G6PD that catalyzes the first step of oxidative branch of PPP is a rate-limiting enzyme of this metabolic pathway. The expression level of G6PD has been reported to increase in various cancer lesions, especially in cancers with advanced stages and metastatic disease, which is associated with poor treatment outcome 18-21.

## Materials and methods

### Cell culture

The cell line MDA-MB-231 was provided by Professor Xiaofeng Le when he was working at the Department of Experimental Therapeutics at the M.D. Anderson Cancer Center, University of Texas. The stable cell line of MDA-MB-231-C3 (231-C3) was generated by transfecting a plasmid DNA which encodes the fluorescence resonance energy transfer (FRET)-based caspase-3 sensor (C3) into MDA-MB-231 cells 22-24. Non-cancerous breast cell line of MCF10A, TNBC cell lines of MDA-MB-157, BT-549 and MDA-MB-468 were purchased from American Type Culture Collection (ATCC, Manassas, VA, USA). MCF10A cells were cultured in MEGM mammary epithelial cell growth medium (Lonza, Alpharetta, GA, USA), while other cells were cultured in DMEM (Thermo Fisher Scientific, Waltham, MA, USA) supplemented with 10% fetal bovine serum (FBS) (Hyclone, Buckinghamshire, UK) and 1% penicillin/streptomycin (Thermo Fisher Scientific, Waltham, MA, USA).

### Determination of cell apoptosis by FRET imaging and cell viability by MTT assay

In each well of a 96-well plate, 5,000 cells were seeded in 100 µL of culture medium. After appropriate treatment, cells were imaged with an inverted fluorescence microscope (Axiovert S100, Carl Zeiss, Germany) equipped with FRET filters (Ex=436±10 nm; diachronic mirror=455 nm; Em_1_=480±20 nm and Em_2_=535±15 nm) and a computer-controlled camera (AxioCam MRm, Carl Zeiss, Germany) which was operated by Zen 2012 software (Carl Zeiss). At least five pictures were captured for each group. The representative pictures were selected and shown. The apoptotic rate was quantified from FRET images using methods described in previous publications 14,22. After FRET imaging, 10 μl of 3-(4,5-dimethylthiazol-2-yl)-2,5-diphenyltetrazolium bromide (MTT, Sigma-Aldrich) solution (5 mg/mL) was added into each well and then the plate was incubated at 37°C for 3 h. After incubation, 100 μl of 10% SDS solution containing 0.01 M HCl was added into each well to solubilize the crystalized formazan. The optical density of the reaction mixture at 595 nm in each well was determined using a plate reader (Perkin-Elmer, USA) after the 96-well plate was incubated at 37°C overnight. IC_50_ was the concentration of DOX inhibiting 50% of the cell growth at 24 h and was determined by GraphPad Prism using a previously described method 25.

### Detection of the intracellular ROS level

To image ROS generation, MDA-MB-231 cells were treated with or without 10 µM DOX in a 60 mm dish for 24 h. Cells were then stained with 10 µM CM-H_2_DCFDA (Thermo Fisher Scientific, Waltham, MA, USA) in FluoroBrite medium (Thermo Fisher Scientific, Waltham, MA, USA) for 20 min. After the incubation, cells were washed with PBS twice and resuspended in FluoroBrite medium for immediate imaging using an inverted fluorescence microscope (Axiovert S100, Carl Zeiss, Germany). At least five pictures were captured for each group. The representative pictures were selected and shown. To quantify ROS level, the CM-H_2_DCFDA-stained cells were washed with PBS twice and resuspended in FluoroBrite medium. The intensity of DCF fluorescence was immediately measured using a LSR Fortessa X-20 flow cytometer (BD, USA).

### Two-dimensional fluorescence difference gel electrophoresis (2D-DIGE) analysis

One dish (10 cm) of 231-C3 or 231-M1 cells in 70% confluence were collected respectively in cold phosphate buffered saline (PBS). Cell pellets were sent to Applied Biomics (Hayward, CA, USA) in dry ice for proteomic analysis using 2D-DIGE, MALDI-TOF and tandem MS analyses. Briefly, cells were lysed with 2D lysis buffer (7 M urea, 2 M thiourea, 4% CHAPS, 30 mM Tris-HCl, pH 8.8) for protein extraction. After measuring and adjusting protein concentration, equal amount of protein sample was labelled with different fluorescent dyes. Protein sample from 231-C3 cells was labeled with Cy2 and that from 231-M1 cells was labeled with Cy3. After labelling, two samples were mixed and about 80 μg of sample was loaded onto one electrophoresis gel (analytical gel) and separated by 2D-DIGE. The 2D gel was scanned using a Typhoon Trio scanner (Amersham BioSciences, Piscataway, NJ). Images were then analyzed by Image Quant software (Amersham BioSciences). All differentially expressed proteins were further analyzed from the 2D-DIGE-derived data using Decyder software (Amersham BioSciences).

A total of 54 spots containing proteins of interest were selected from the analytical gel. The selection criteria were that Cy3/Cy2 ratio is either more than 1.5 or less than -1.5. For protein identification, about 600 μg of unlabeled protein and a small amount of labeled proteins were further loaded and separated in a preparation 2D gel. The proteins of interest were picked using a fully automatic Ettan Spot Picker. The picked samples were washed multiple times to remove staining dyes and other chemicals. After digestion with trypsin buffer in gel at 37°C, protein samples were extracted, desalted, mixed with α-cyano-4-hydroxycinnamic acid matrix and were subjected to MALDI-TOF MS/MS analysis (Applied Biosystems 4700 Proteomics Analyzer, Applied Biosystems, Foster City, CA). All identified 54 protein IDs were further analyzed by Panther Classification System based on molecular function, protein class and biological process.

### Western blot analysis

The cells with or without treatments were collected and lysed with RIPA buffer containing protease inhibitors and phosphatase inhibitors (Sigma-Aldrich, Saint Louis, MO, USA). The protein concentration was determined using a Bradford assay kit (Bio-Rad, Berkeley, CA, USA). Proteins in cell lysate were separated by SDS-PAGE and transferred to a PVDF membrane. After blocking with 5% low-fat milk, the membranes were incubated with primary antibodies of G6PD (dilution of 1:000) or β-actin (dilution of 1:5000) overnight at 4 °C. The protein blots were further probed with HRP-conjugated secondary antibodies (dilution of 1:5000) for 1 h at room temperature and developed in ECL solution (Thermo Scientific, USA). The intensities of the bands on the membrane were measured using Image Pro Plus software (Media Cybernetics, USA).

### Clinical data analysis

The expression data of G6PD in clinical samples was taken from the study by Curtis et al (2012) 26. The dataset was generated by transcriptional profiling on the Illumina HT-12 v3 platform, followed by normalization. It is stored on European Genome-Phenome Archive (EGA, http://www.ebi.ac.uk/ega/), under accession number EGAS00000000083. The clinical annotations for normal samples and TNBC samples were provided in the Supplementary Information from the same study. The boxplot was generated in R using ggplot2 package. Welch's *t*-test for comparing the mean of G6PD expression between normal and TNBC samples was performed using ggpubr package.

Clinical follow-ups and G6PD expression data were obtained from the same aforementioned study. The R packages survival and survminer were used to generate the Kaplan-Meier plot. Patients were divided into G6PD high and low expression groups by applying a cutoff of 60 percent quartile on normalized expression values. Mantel-Haenszel logrank tests were used to compare the overall survival probabilities of two subgroups of TNBC patients and to compute R-value.

### Knockdown and inhibition of G6PD

Validated Silencer Select siRNA against G6PD (siRNA ID s5447) and Silencer Select Negative Control siRNA were purchased from Thermo Fisher Scientific (Waltham, MA, USA). Cells were seeded into 60-mm dishes for overnight culture before transfection. After washing twice with PBS, cells were transfected with 20 nM siRNA in OptiMEM (Life Technologies) using Lipofectamine RNAiMAX (Thermo Fisher Scientific), following the manufacturer's instructions. After a 6-h incubation, the transfection complex was replaced with regular complete DMEM. After 45 h, the cells were subjected to further experiments. To inhibit the activity of G6PD, 150 or 200 μM of DHEA (Sigma-Aldrich) was used to pre-treat the cells for 12 h before they were subjected to further treatment. DHEA continuously existed for the whole duration of compound treatment.

### Detection of intracellular DOX concentration

Cells were seeded in 60-mm dishes and then incubated in phenol-red free DMEM containing 5 μM DOX for different time periods. After DOX treatment, cells were washed twice in ice-cold PBS and harvested. Cell pellets were obtained by centrifugation at 800 *g* for 3 min at 4 °C. Cells were then lysed in RIPA buffer and subjected to sonication. A small portion of cell lysate was measured for cellular protein concentration. The remaining cell lysate was mixed with acidified isopropanol (0.75 M HCl) (v/v 1:3) to extract DOX. After thorough vortexing and overnight incubation at -20 °C, the mixture was centrifuged at 15,000 *g* for 10 min. Fluorescence intensity of the organic phase was determined by a spectrofluorometer (SpectraMax M5, Molecular Devices). Excitation and emission wavelengths were set at 480 nm and 590 nm, respectively. Fluorescence intensity was converted to the amount of DOX using a standard curve. The amount of DOX (nM) was normalized to the amount of cellular protein (mg).

### Reduced GSH level determination

Cells with or without G6PD silencing were grown in 60-mm dishes. After 5 μM DOX treatment for different time periods, cells were collected and lysed in PBS with 0.5% NP-40. The reduced GSH level was determined using the GSH/GSSG ratio detection assay kit (ab138881, Abcam, USA). The excitation and emission wavelengths of the fluorescence were set at 490 nm and 520 nm, respectively, and their intensity was determined by using a spectrofluorometer (SpectraMax M5, Molecular Devices).

### Statistical analysis

All data were represented as the mean ± SD of at least three independent experiments. Statistical significance was analyzed using Student's *t*-test for comparison between two groups, one-way ANOVA followed by *t*-test for multiple groups, and two-way ANOVA followed by *t*-test for multiple groups with two parameters. *p < 0.05, **p < 0.01, and ***p < 0.001.

## Results

### Metastatic TNBC cells develop resistance to DOX treatment that induces high level of intracellular oxidative stress

**Figure 1:**
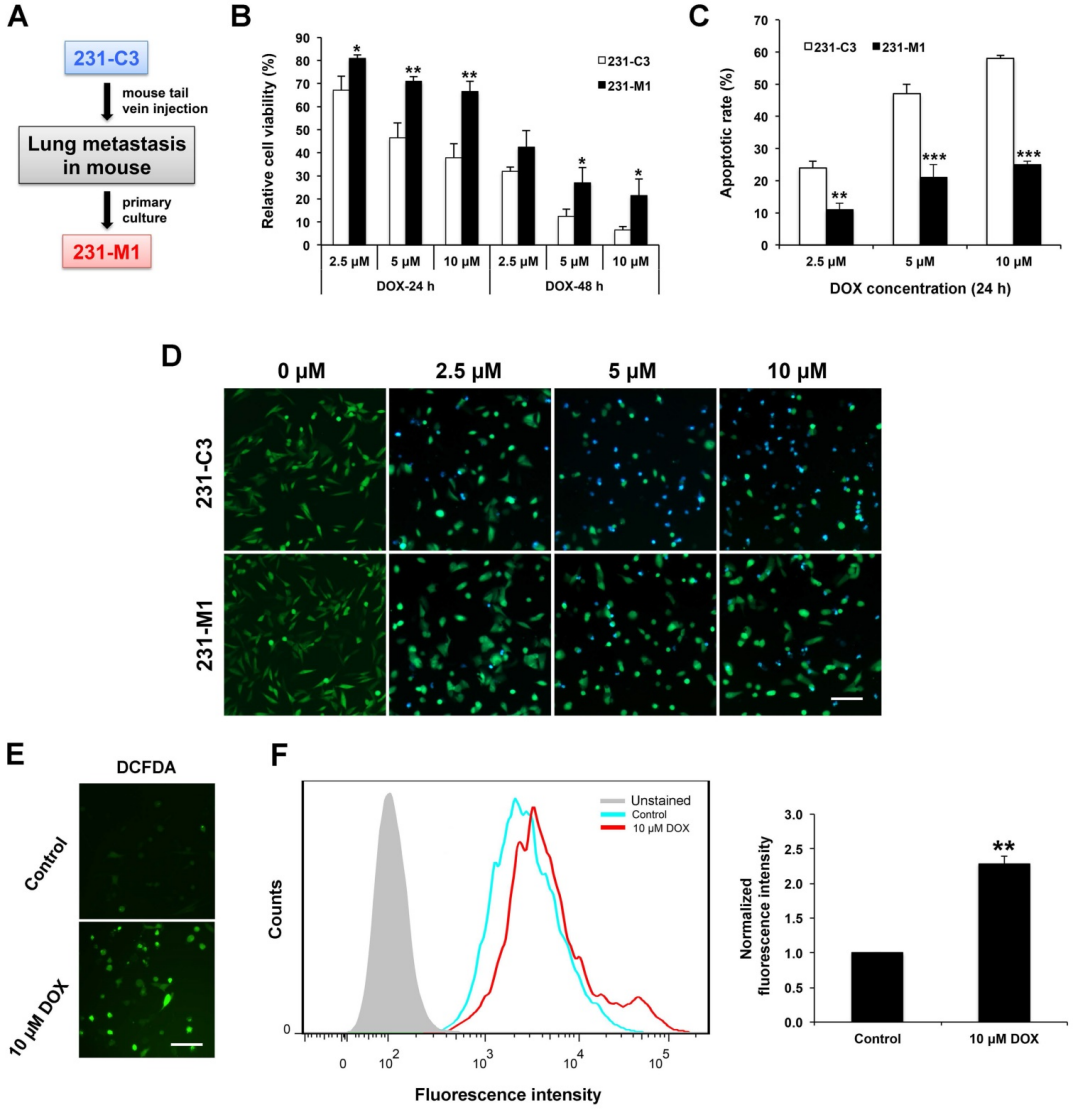
** Metastatic 231-M1 cells develop resistance to DOX treatment that induces high level of intracellular oxidative stress. (A)** Schematic diagram of 231-M1 cells generation. 231-C3 cells were injected into tail vein of a nude mouse. After 45 days, a micrometastasis was formed in the lung of the mouse and dissected for primary culture and a novel metastatic TNBC cell line, 231-M1 was generated. **(B)** 231-M1 cells displayed significantly higher cell viability than 231-C3 cells upon DOX treatment. The cells were treated with DOX at 2.5 µM, 5 µM, and 10 µM respectively for 24 h, 48 h and subjected to MTT assay. The results were normalized to the control group without drug treatment. (C-D) 231-M1 cells exhibited significantly lower apoptotic rate than 231-C3 cells upon DOX treatment. The cells were treated with DOX at 2.5 µM, 5 µM, and 10 µM respectively for 24 h with representative FRET images were shown in (D). Apoptotic rate of these sensor cells was quantified by FRET imaging, as healthy cells exhibited green color while apoptotic cells turned to blue color. Scale bar, 100 µm. (E) MDA-MB-231 cells were treated with or without 10 µM (Control) of DOX for 24 h and stained with a fluorescent dye (CM-H_2_DCFDA) for detecting ROS. The representative fluorescent images show that DOX treatment produced a high level of ROS in MDA-MB-231 cells. Scale bar, 100 µm. (F) The left panel shows the flow cytometry analysis of these stained cells and cells without staining (Unstained). Right panel shows the quantified fluorescence intensity from the flow cytometry analysis. The results were normalized to DCFDA-stained control group without DOX treatment.

We previously established an engineered TNBC cell line, 231-C3, that expresses a fluorescent apoptosis sensor. The 231-C3 cells appear green when alive and blue when undergo apoptosis due to the reduction of fluorescence resonance energy transfer (FRET) between the donor, cyan fluorescent protein, and the acceptor, yellow fluorescent protein of this apoptosis sensor. To generate a lung metastatic TNBC cell line, 231-C3 cells were injected into tail vein of a nude mouse. After 45 days, a micrometastasis on the lung of the mouse was dissected for primary culture, then the cells were isolated and cultured into a cell line named as 231-M1, which exhibited stronger metastatic ability than parental 231-C3 cells 14 (Figure 1A). Both 231-C3 and 231-M1 cells displayed significant difference when they were treated with DOX *in vitro*. The 231-C3 cells displayed a dose and time dependent response to DOX treatment (Figure 1B) and when the concentration was above 5 μM, obvious cell apoptosis was triggered as revealed by FRET imaging (Figure 1D). Compared with 231-C3 cells, 231-M1 cells are less sensitive to DOX treatment, exhibiting significantly higher cell viability and less cell apoptosis (Figure 1B and D). Following 5 μM and 10 μM DOX treatments for 24 h, the viability of 231-M1 cells was 71% and 67%, respectively, while the viability of 231-C3 cells were 46% and 38%, respectively. When treatment time was extended to 48 h, 231-M1 cells exhibited more than 2-fold higher cell viability compared to that of 231-C3 cells upon DOX treatment at 5 μM and 10 μM (Figure 1B). We then quantified the apoptotic rate based on FRET imaging. The results show that only 11%, 21%, and 25% of 231-M1 cells underwent apoptosis after 24 h of DOX treatment at 2.5 μM, 5 μM, and 10 μM, respectively. In contrast, 231-C3 cells showed much higher apoptotic rate of 47-58% at the same concentrations of DOX (Figure 1C and D).

To elucidate the development of higher drug resistance towards DOX treatment of 231-M1 cells compared to 231-C3 cells, we investigated the mechanism of action of DOX. Many studies suggested DOX could induce high level of oxidative stress inside cells. In this study, a general oxidative stress indicator, CM-H_2_DCFDA, was used to detect oxidative stress level inside the cells during DOX treatment. Oxidation of this indicator inside cells leads to the increase of green fluorescence that can be imaged by fluorescence microscope and quantified by flow cytometry. The images in Figure 1E show that MDA-MB-231 cells emitted strong green fluorescence after they were treated with 10 μM DOX for 24 h compared with untreated control cells. The quantitative flow cytometry results presented a significant 2.3-fold increase in fluorescence intensity from the untreated control to DOX treated cells (Figure 1F). These results indicate that a high level of intracellular oxidative stress was generated after cells were treated with DOX.

### G6PD is up-regulated in 231-M1 cells compared with 231-C3 cells

**Figure 2:**
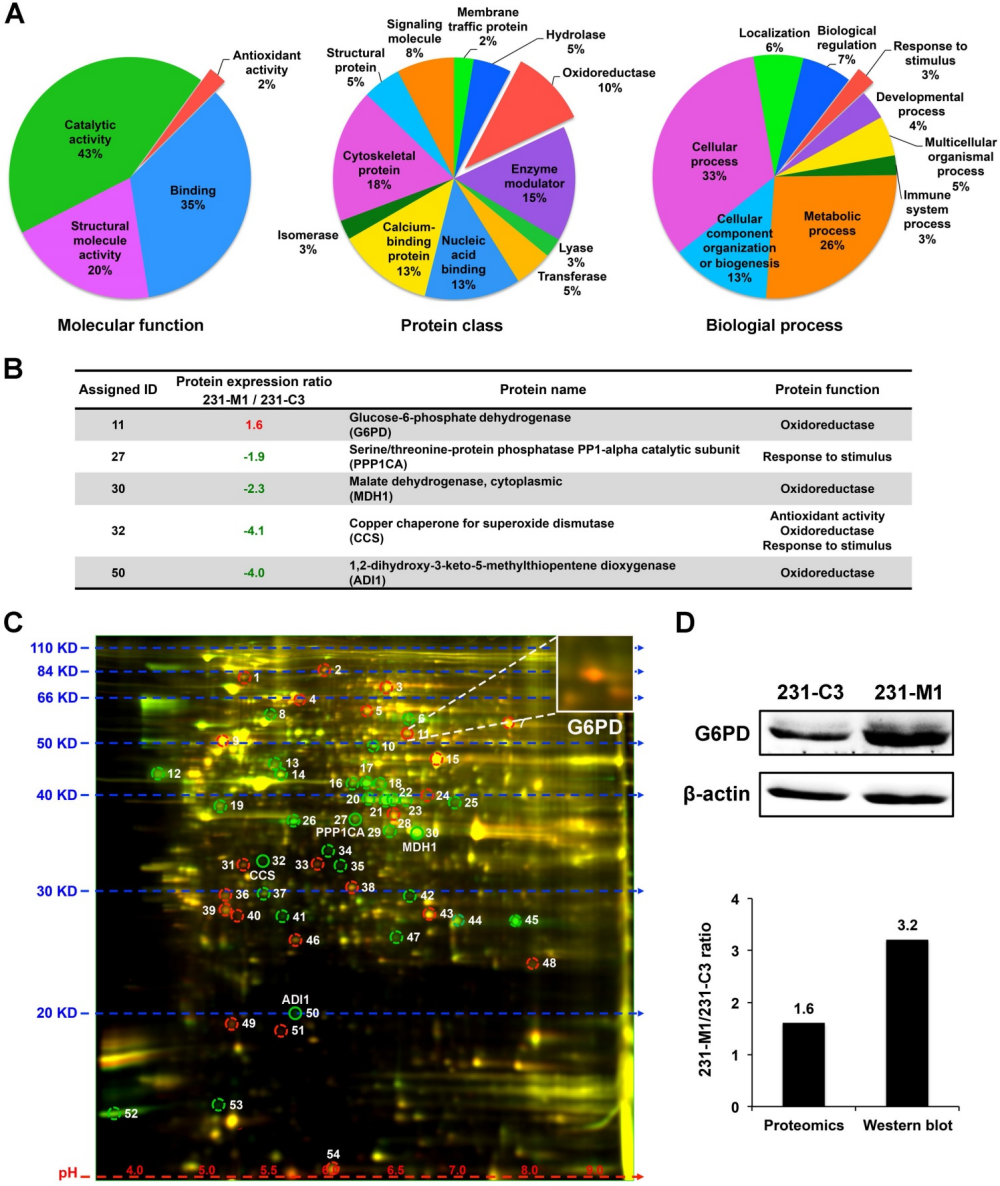
** G6PD is up-regulated in 231-M1 cells compared to 231-C3 cells. (A)** Classification of total 54 differentially expressed proteins that were identified through 2D-DIGE proteomics by online Panther system. Three groups of proteins (antioxidant activity, oxidoreductase and response to stimulus) were selected out. **(B)** The information of five proteins that belong to aforementioned three groups are summarized in a table. G6PD is the only protein that is up-regulated in 231-M1 cells compared with 231-C3 cells. **(C)** Fluorescent image of 2D-DIGE proteomics gel. G6PD was assigned with ID 11 and was shown as an orange spot on the gel that was enlarged in the upper right corner of the image. **(D)** The result of G6PD high expression from the proteomics analysis was validated by Western blot analysis. Upper panel shows Western blot analysis of the G6PD and β-actin levels in 231-C3 and 231-M1 cells. Lower panel shows the quantified ratio of G6PD in 231-M1 to 231-C3 cells in proteomics and Western blot result.

Because 231-M1 cells survived better than 231-C3 cells under DOX treatment that induced high level of oxidative stress inside the cells, we hypothesized that 231-M1 cells might have higher antioxidant ability over 231-C3 cells. To identify possible differentially expressed antioxidant proteins between these two cell lines, we compared the protein profiles of 231-C3 and 231-M1 cells using quantitative 2D-DIGE proteomics analysis. Fifty-four differentially expressed proteins were identified. These proteins were classified through online Panther classification system for molecular function, protein class and biological process. We focused on three groups of proteins: proteins with antioxidant ability that can reduce oxidative stress directly (2% in left panel of Figure 2A, colored in red), the oxidoreductase that catalyzes redox reactions in cells thus probably facilitates intracellular antioxidant molecules generation (10% in middle panel of Figure 2A, colored in red) and proteins that are responsive to stimulus (3% in right panel of Figure 2A, colored in red).

There are five proteins in total in these three groups, among them an oxidoreductase, G6PD, is the only one that was significantly up-regulated in 231-M1 cells compared with 231-C3 cells. The other four proteins were down-regulated in 231-M1 compared with 231-C3 cells (Figure 2B). The assigned ID number of G6PD was 11 and it was shown on the gel as an orange color spot (Figure 2C), indicating that 231-M1 cells have higher G6PD level than 231-C3 cells. The increase of G6PD level from 231-C3 to 231-M1 cells was confirmed by Western blot analysis. The quantified ratio of G6PD between 231-M1 and 231-C3 cells was 1.6 by 2D-DIGE proteomics analysis and 3.2 by Western blotting (Figure 2D).

### High G6PD expression is correlated with poor DOX response in TNBC cells and is associated with low overall survival rate of TNBC patients

**Figure 3:**
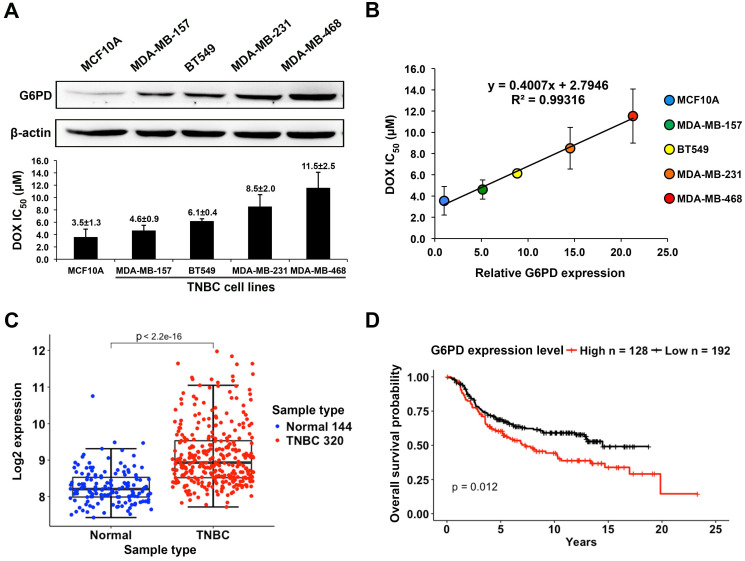
** High G6PD expression is positively correlated with poor DOX response in TNBC cells and is associated with low overall survival rate of TNBC patients. (A)** Upper panel shows the Western blot result of G6PD level in MCF10A and four TNBC cell lines including MDA-MB-157, BT549, MDA-MB-231, and MDA-MB-468. Lower panel shows IC_50_ values of DOX in these five cell lines calculated from MTT assay results. **(B)** Linear regression analysis of G6PD expression level and DOX IC_50_ values of these cell lines revealed a strong positive correlation with a R-square of 0.993. **(C)** The comparison of G6PD expression level between TNBC and normal tissues. Data is taken from Curtis et al. (2012), and sample size is indicated in each type. **(D)** The overall survival probabilities of TNBC patients with high and low G6PD expression in Curtis et al (2012). P-value was calculated using Mantel-Haenszel logrank test.

The G6PD expression level was examined by Western blot analysis in the human mammary epithelial cell line MCF10A and four TNBC cell lines including MDA-MB-157, BT549, MDA-MB-231, and MDA-MB-468. These cells were treated with DOX at various concentrations for 24 h and the IC_50_ (µM) of DOX in each cell line was measured (Figure 3A). The result showed a strong positive correlation between the relative levels of G6PD and the values of DOX IC_50_ with the R^2^ value of 0.993 (Figure 3B). In particular, MCF10A cells that have a very low level of G6PD were sensitive to DOX with a low IC_50_ of 3.5±1.3 μM, while high G6PD-expressing MDA-MB-468 cells were less responsive to DOX with a high IC_50_ of 11.5±2.5 μM.

By analyzing the breast cancer dataset reported by Curtis et al, we found that the average G6PD expression level in TNBC is higher than that in normal tissue (Welch's *t*-test, p value < 2.2×10^-16^) (Figure 3C), which further indicates a functional importance of G6PD in supporting the survival of TNBC cells. Moreover, the overall survival analysis revealed that TNBC patients with a higher G6PD expression level tended to have a lower survival probability compared to those with a relatively low G6PD expression level (Figure 3D). The data further supports the possibility that G6PD expression level in TNBC is positively correlated with DOX-resistance degree of TNBC cells.

### Downregulating G6PD expression reduces cell viability and increases apoptotic rate of TNBC cells upon DOX treatment

**Figure 4:**
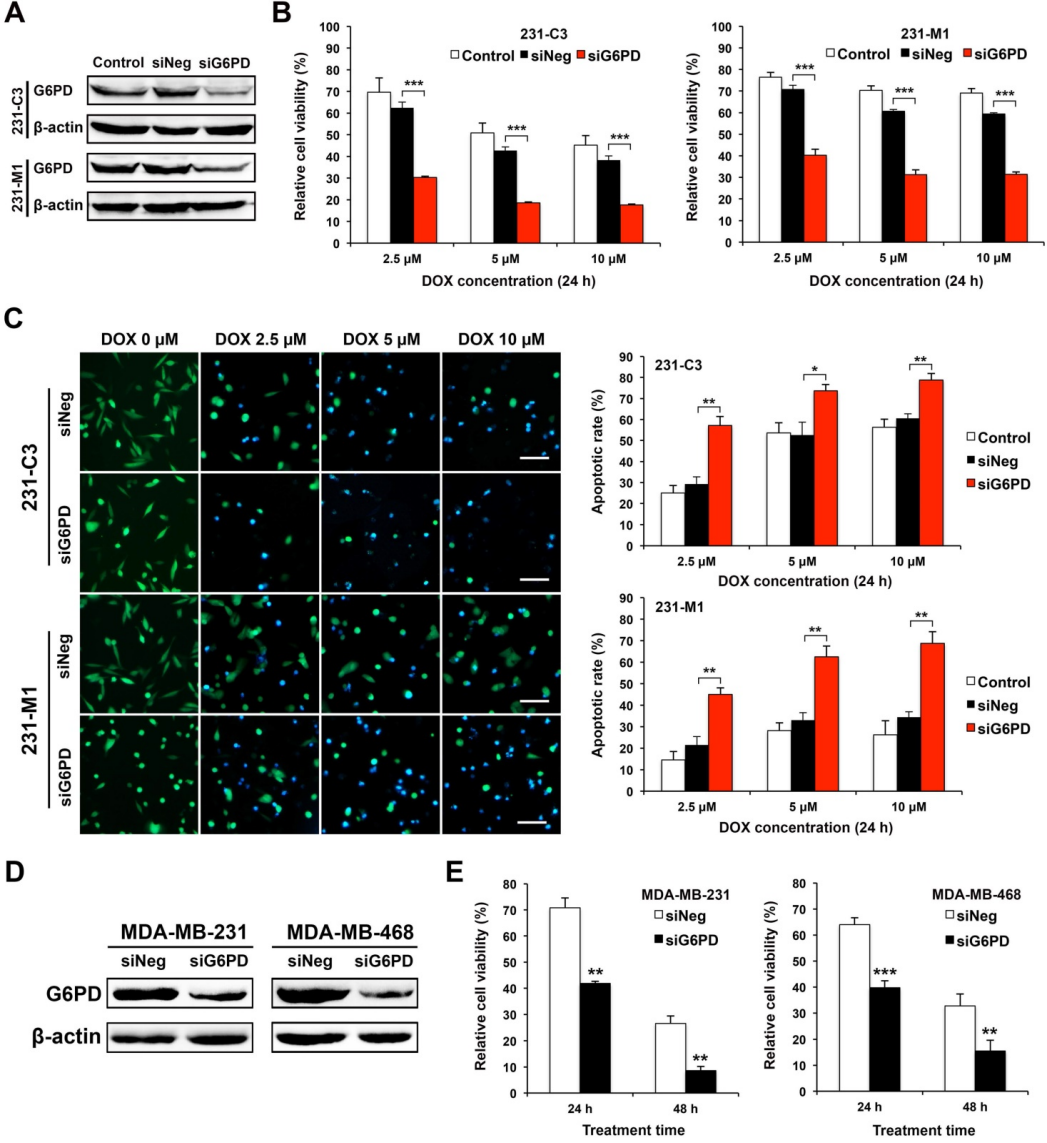
** Downregulating G6PD expression reduces cell viability and increases apoptotic rate of TNBC cells upon DOX treatment. (A)** Western blot results of G6PD levels in 231-C3 and 231-M1 cells that were not transfected or transfected with 20 nM of siNeg (siRNA negative control) or siG6PD. **(B)** G6PD silencing by siRNA decreased cell viability of both 231-C3 and 231-M1 cells upon DOX treatment. After 45 h post siRNA transfection, 231-C3 or 231-M1 cells were treated with DOX at 2.5 µM, 5 µM, and 10 µM respectively for 24 h. The results were normalized to viability of cells without DOX treatment nor siRNA transfection. **(C)** G6PD silencing by siRNA increased apoptotic rates of both 231-C3 and 231-M1 cells. Left panel shows representative FRET images of 231-C3 and 231-M1 cells with either siNeg or siG6PD treatment that were further subjected to DOX treatment at 0 µM, 2.5 µM, 5 µM, and 10 µM respectively for 24 h. Scale bar, 100 µm. Right panels show apoptotic rates of 231-C3 and 231-M1 cells that were quantified using at least 300 cells from more than five FRET images. **(D)** Western blot analysis of G6PD levels in MDA-MB-231 and MDA-MB-468 cells that were transfected with 20 nM of either siNeg or siG6PD. **(E)** G6PD silencing by siRNA decreased cell viability of both MDA-MB-231 and MDA-MB-468 cells. After 45 h post siRNA transfection, the cells were treated with DOX at 5 µM for either 24 h or 48 h before subjected to MTT assay. The results were normalized to the control group without DOX treatment.

The expression of G6PD was significantly reduced by gene specific siRNA-mediated silencing in both 231-C3 and 231-M1 cells (Figure 4A). Cells transfected with negative control siRNA (siNeg) or siG6PD or without siRNA transfection (control) were treated with DOX at 2.5 μM, 5 μM, and 10 μM for 24 h. MTT results show that the cells with G6PD knockdown exhibited significantly lower cell viability after 24 h of DOX treatment compared with cells in the Control and siNeg group. Specifically, after 24 h of DOX treatment at 2.5 μM, 5 μM, and 10 μM, the relative cell viability of 231-C3 cells with G6PD silencing is 30%, 19%, and 18%, respectively, which is much less than that of the siNeg group of 62%, 43%, and 38%, respectively (Figure 4B, left graph).

Similar results were also observed in 231-M1 cells, despite their higher G6PD level. After 24 h of DOX treatment at 5 μM and 10 μM, the G6PD silenced cells exhibited a 31% cell viability, which was almost half of the cell viability in the siNeg transfected cells (61% at 5 μM and 59% at 10 μM respectively) (Figure 4B, right graph).

The decreased cell viability after G6PD silencing was due to the increased apoptosis induced by DOX. As shown by the FRET images (Figure 4C, left panel), after 24 h of DOX treatment, more apoptotic cells were observed in 231-M1 cells treated with siG6PD at all three concentrations of DOX (2.5 μM, 5 μM, and 10 μM). For 231-C3 cells, as apoptotic rates of above 50% in both control and siNeg transfected cells upon 24 h DOX treatment at 5 μM and 10 μM were already high, silencing G6PD only further increased the apoptotic rate by about 20% under the same treatment condition (Figure 4C, right graph). In contrast, for 231-M1 cells without G6PD suppression, low apoptotic rates of 15-28% were detected for 24 h DOX treatment at the concentrations of 2.5 μM, 5 μM, and 10 μM. Notably, when G6PD was suppressed by siRNA, the apoptotic rates of 231-M1 exhibited 2.2-3-fold increases to 45%, 63%, and 69% respectively for these three DOX concentrations (Figure 4C, right graph).

We also validated these results with wild type MDA-MB-231 cells and another TNBC cell line, MDA-MB-468. Silencing G6PD successfully reduced the overall level of G6PD in these two TNBC cell lines (Figure 4D). The G6PD reduction in MDA-MB-231 cells decreased cell viability to 40% after 24 h and 9% after 48 h of 5 μM DOX treatment. In MDA-MB-468 cells, the G6PD suppression further decreased cell viability by 25% after 24 h and 20% after 48 h of 5 μM DOX treatment (Figure 4E). Notably, for both MDA-MB-231 and MDA-MB-468 cells, the viability of G6PD-silenced cells after 48 h of DOX treatment was less than half of the siNeg-silenced cells (Figure 4E).

### Inhibiting G6PD activity with its inhibitor DHEA sensitizes TNBC cells to DOX treatment

**Figure 5:**
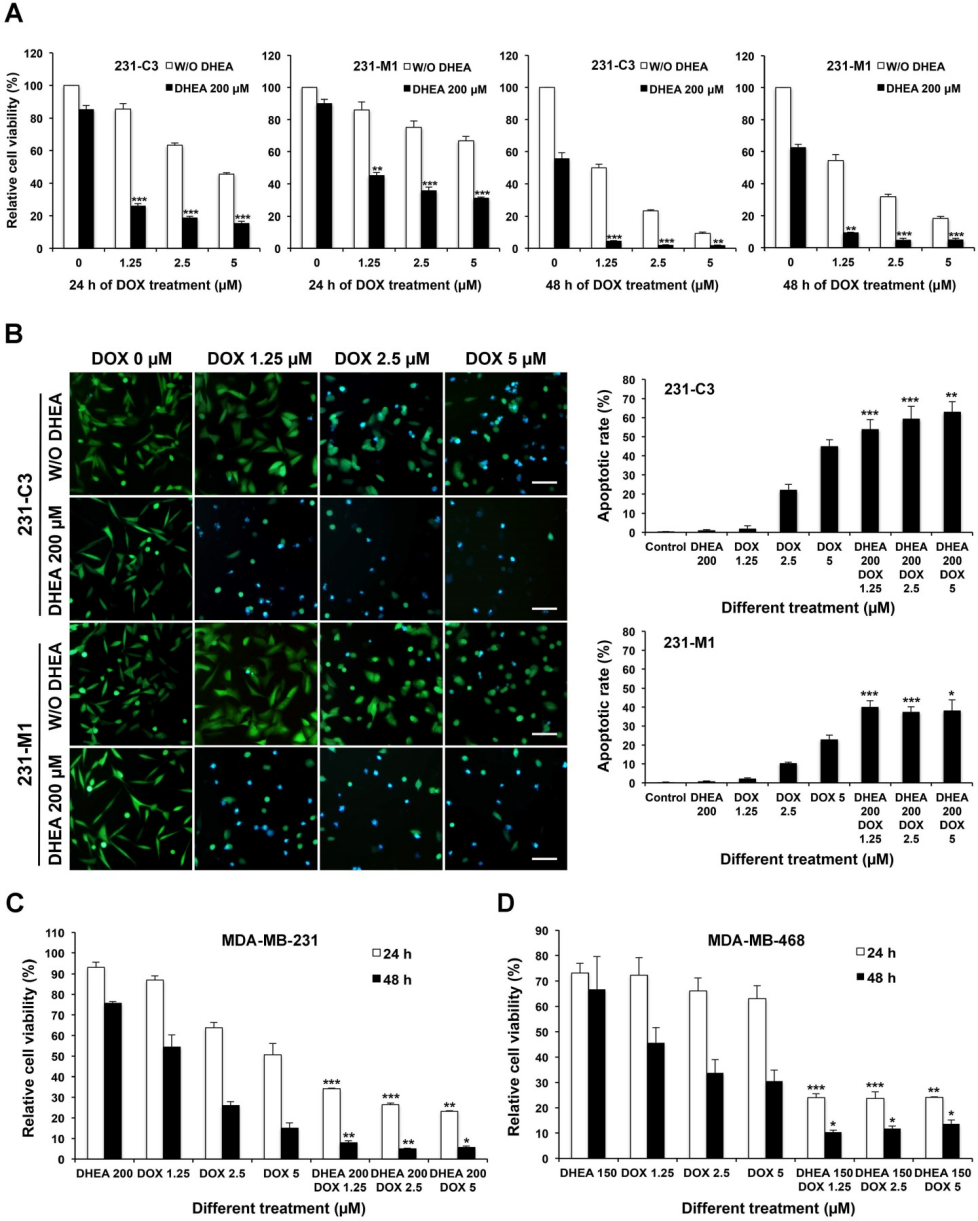
** Inhibiting G6PD activity by its inhibitor DHEA sensitizes TNBC cells to DOX treatment. (A)** 231-C3 or 231-M1 cells were pretreated with or without 200 µM DHEA for 12 h and then cells were subjected to a combinational treatment of 200 µM DHEA with DOX at 0 µM, 1.25 µM, 2.5 µM, or 5 µM respectively for a further 24 h or 48 h. After the treatment, cell viability was determined by MTT assay and normalized to cells without any treatment. **(B)** G6PD inhibition by DHEA significantly increased apoptotic rates of 231-C3 and 231-M1 cells upon DOX treatment. 231-C3 or 231-M1 cells with or without 12 h of 200 µM DHEA pretreatment were subjected to the combinational treatment of 200 µM DHEA with DOX at 0 µM, 1.25 µM, 2.5 µM, or 5 µM respectively for a further 24 h. Left panel shows representative FRET images of 231-C3 and 231-M1 cells under different concentrations of DHEA and DOX. Scale bar, 100 µm. Right panel shows apoptotic rates of 231-C3 and 231-M1 cells that were quantified using at least 300 cells from more than five FRET images. **(C)** MTT assay result of MDA-MB-231 cells with or without 12 h of 200 µM DHEA pretreatment before subjected to the combinational treatment of 200 µM DHEA with DOX (1.25 µM, 2.5 µM, or 5 µM respectively) or DOX treatment alone for a further 24 h or 48 h. The results were normalized to the control group without any treatment. **(D)** MTT assay result of MDA-MB-468 cells with or without 12 h of 150 µM DHEA pretreatment before subjected to the combinational treatment of 150 µM DHEA with DOX (1.25 µM, 2.5 µM, or 5 µM respectively) or DOX treatment alone for a further 24 h or 48 h. The results were normalized to the control group without any treatment. For each DOX concentration, the significant difference was determined between cells treated without or with DHEA.

Inhibition of G6PD activities in 231-C3 and 231-M1 cells was achieved by pretreatment with 200 µM DHEA, which is an inhibitor of G6PD for 12 h. Following which, the cells were treated with both 200 µM DHEA and DOX at 1.25 µM, 2.5 µM, and 5 µM, respectively for another 24 h or 48 h. Compared with cells treated with DOX alone, cells treated with DHEA and DOX together exhibited significantly lower cell viability as examined by MTT assay (Figure 5A). Upon inhibition of G6PD by DHEA, the cell viability of 231-C3 cells reduced from around 80%, 60%, and 45% to around 20% after 24 h of DOX treatment at 1.25 µM, 2.5 µM, and 5 µM respectively (Figure 5A). The viability of 231-M1 cells also showed about 40% reduction when they were treated with DOX and 200 µM DHEA together (Figure 5A). Interestingly, when the treatment time was extended to 48 h, the cell viability of 231-C3 dramatically decreased to less than 5% and that of 231-M1 was also decreased to less than 10% in all three DOX concentrations. Notably, when cells were treated with 200 µM DHEA alone for overall 36 h or 60 h, the viabilities of both 231-C3 and 231-M1 cells were above 90% and above 55%, respectively, suggesting that DEHA has little cytotoxicity within 36 h of treatment time (Figure 5A).

The apoptotic rates of 231-C3 and 231-M1 cells after 24 h of DOX treatment with or without G6PD inhibition by 200 µM of DHEA were revealed by FRET imaging. DHEA alone at a concentration of 200 µM did not induce any apoptosis in both 231-C3 and 231-M1 cells. Pretreatment with 200 µM of DHEA for 12 h significantly sensitized 231-C3 and 231-M1 cells to DOX-induced apoptosis (Figure 5B, left panel). In particular, when 231-C3 cells were treated with DOX alone at 1.25 µM, 2.5 µM, and 5 µM for 24 h, the apoptotic rates were around 2%, 22%, and 44%, respectively. It is to be noted that the combinational treatment of DHEA and DOX for 24 h increased the apoptotic rates to 54-62% for all three DOX concentrations (Figure 5B, right graphs). In 231-M1 cells, the apoptotic rates with DOX treatment alone at 1.25 µM, 2.5 µM, and 5 µM were 2%, 10%, and 23%, respectively, which were increased to around 40% for all three DOX concentrations when cells were treated together with DHEA (Figure 5B, right graphs).

Notably, there was very little apoptosis detected in 231-C3 and 231-M1 cells upon either 200 µM DHEA or 1.25 µM DOX treatment alone. In strong contrast, the apoptotic rates were dramatically increased to 54% and 40% in 231-C3 and 231-M1 cells respectively when these cells were co-treated with 200 µM DHEA and 1.25 µM DOX together (Figure 5B). Similar results were also obtained in wild type TNBC, MDA-MB-231 and MDA-MB-468 cells. Combinational treatment with DHEA and DOX significantly reduced cell viability of MDA-MB-231 (Figure 5C) and MDA-MB-468 cells (Figure 5D). Especially in 48 h of combinational treatment, the viability of MDA-MB-231 cells was reduced to less than 10% in all three concentrations, which was in a sharp contrast to the cell viability in 48 h-DOX treatment alone (53% at 1.25 µM) (Figure 5C). In MDA-MB-468 cells, combination of DHEA with 1.25 µM, 2.5 µM, and 5 µM DOX also decreased cell viability from 31-45% to 10-12% in all three DOX concentrations (Figure 5D). These results indicate a pharmaceutical potential of using a combination of G6PD inhibitor DHEA with a low concentration of DOX to treat TNBC tumors that are resistant or non-responsive to DOX treatment.

### G6PD enables TNBC cells to maintain a relatively high intracellular GSH level during DOX treatment

**Figure 6:**
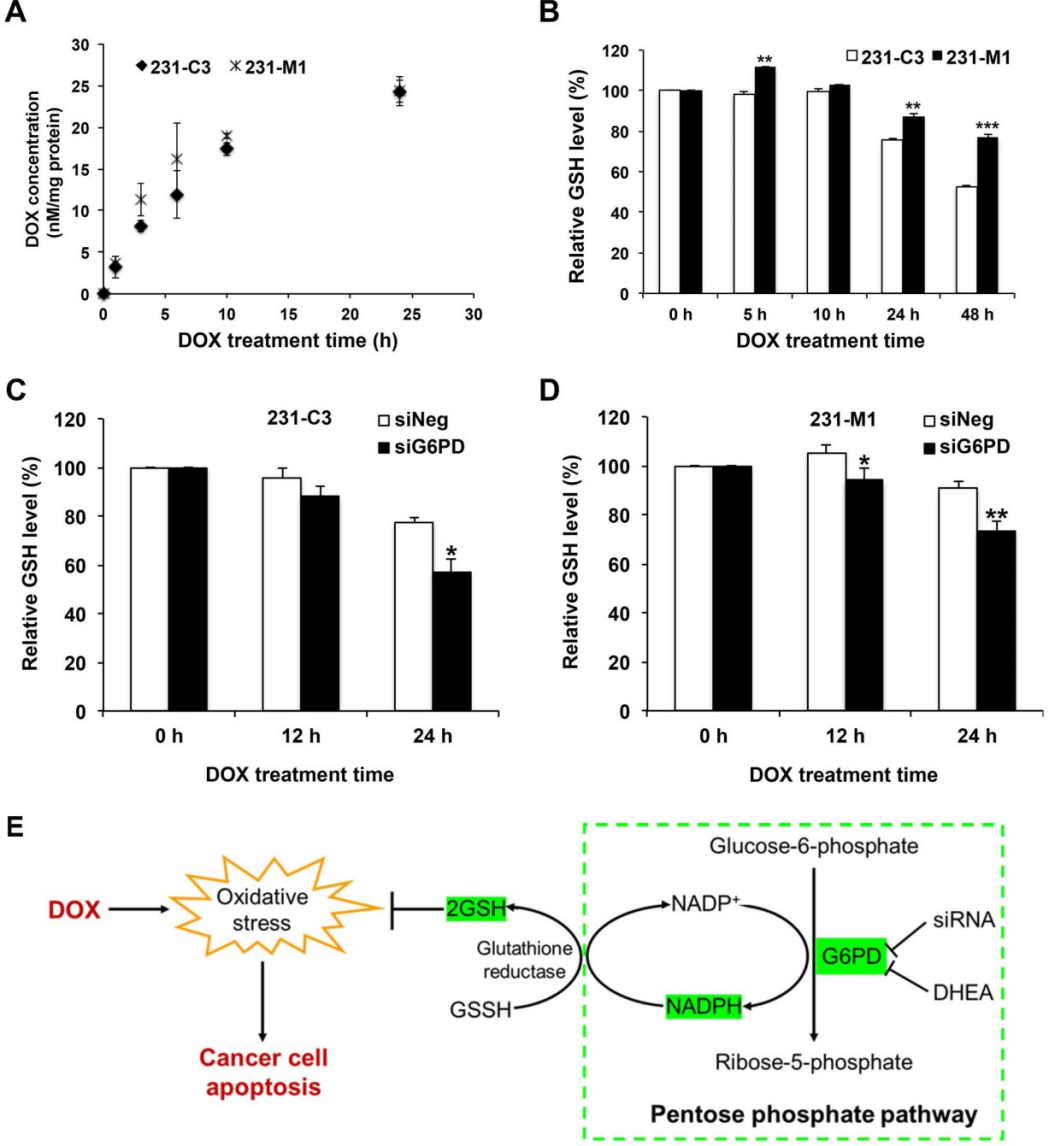
** G6PD enables TNBC cells to maintain a relatively high intracellular GSH level during DOX treatment. (A)** Intracellular DOX concentration of 231-C3 and 231-M1 cells upon 5 µM DOX treatment for 0, 1, 3, 6, 10, and 24 h respectively. The intracellular level of DOX was normalized to the amount of proteins of the cells. **(B)** Relative reduced intracellular GSH level of 231-C3 and 231-M1 cells upon 5 µM DOX treatment at 0, 5, 10, 24, and 48 h respectively. GSH levels were normalized to 231-C3 level at 0 h of DOX treatment. **(C)** 231-C3 cells were transfected with either negative or G6PD-specific siRNA before DOX treatment. GSH levels of 231-C3 cells upon 5 µM DOX treatment at 0, 12, and 24 h respectively were measured and normalized to GSH level at 0 h of DOX treatment. **(D)** Relative GSH level inside 231-M1 cells upon 5 µM DOX treatment at 0, 12, and 24 h respectively after 231-M1 cells were transfected with either negative or G6PD specific siRNA. GSH levels were normalized to GSH level at 0 h of DOX treatment. **(E)** Schematic showing the role of G6PD in pentose phosphate pathway to produce NADPH, GSH and the mechanism of high level of G6PD in conferring DOX resistance in TNBC cells.

To further understand why metastatic 231-M1 cells with higher G6PD expression survived better than 231-C3 cells upon DOX treatment, the mechanism of G6PD-mediated DOX-resistance was investigated. Intracellular DOX concentration was first measured to check whether the differential DOX response between 231-C3 and 231-M1 cells was due to differential drug uptake or multidrug efflux pumps. When cells were treated with DOX at 5 µM, the intracellular DOX concentration of 231-C3 and 231-M1 cells was detected and calculated based on the red fluorescence emitted from DOX. The result shows that the drug uptake curves were similar between 231-C3 and 231-M1 cells without statistical differences, which excludes the decreased drug uptake as a major mechanism of DOX resistance in metastatic 231-M1 cells (Figure 6A).

GSH regulation was hypothesized to be the most probable mechanism of G6PD-mediated DOX resistance, because GSH is a major antioxidant molecule inside cells and it can protect cells from apoptosis triggered by DOX-generated ROS 27-29. In this study, the GSH level was measured in 231-C3 and 231-M1 cells during 5 µM DOX treatment for up to 48 h. The result of 231-C3 cells shows that DOX treatment could exhaust the GSH pool after 24 h by reducing GSH level (Figure 6B). In contrast, the GSH level in 231-M1 cells was relatively stable over DOX treatment compared to 231-C3 cells (Figure 6B). In particular, DOX treatment reduced the intracellular GSH level by 24% and 47% in 231-C3 cells after 24 h and 48 h of treatment, respectively, which was consistent with the increased apoptotic rate of 231-C3 cells after DOX treatment (Figure 1B). In 231-M1 cells, the GSH reduction was only 13% after 24 h and 23% after 48 h of DOX treatment (Figure 6B), which is consistent with a higher viability and lower apoptotic rate detected from 231-M1 cells (Figure 1B and C) compared with 231-C3 cells.

Decreasing G6PD level by siRNA transfection further exhausted the level of GSH in 231-C3 cells and also affected the relatively stable GSH level in 231-M1 cells. As shown in Figure 6C, when G6PD expression was suppressed, the intracellular GSH level of 231-C3 cells was reduced from 77% to 57% after 24 h DOX treatment at 5 µM. DOX treatment also reduced the GSH level in 231-M1 cells for a further 20% in G6PD-silenced cells compared to siNeg cells after 24 h of 5 µM DOX treatment (Figure 6D). Overall, these results suggest that G6PD could enable TNBC cells to maintain a relatively stable intracellular GSH level which could be used to scavenge the ROS generated during DOX treatment.

## Discussion

Several reports have elucidated the mechanisms of DOX resistance in cancer cells such as up-regulation of multidrug resistance protein 1 (also known as P-glycoprotein), high-expression of anti-apoptotic proteins of B-cell lymphoma 2 (Bcl-2) and B-cell lymphoma-extra-large (Bcl-xL), down-regulation or mutation of topoisomerase II, decreased apoptosis caused by p53 mutation and altered proliferation of cancer cells 30-34. Although several studies have reported that DOX can generate oxidative stress 8-11, there are few research reported the relationship between DOX resistance of cancer cells and their antioxidant ability. A previous study in our lab has demonstrated that knockdown of MnSOD, a mitochondrial antioxidant protein, significantly sensitized TNBC cells to DOX-induced apoptosis, likely due to the reduction of antioxidant ability 14.

Inspired by our previous study, we attempted to explore other possible antioxidant proteins that can protect TNBC cells from oxidative stress induced by DOX and to discover possible therapeutic targets for reducing DOX resistance. Through 2D-DIGE proteomics study, we identified G6PD as a potential cause of DOX resistance. Either reduction of G6PD expression by siRNA or inhibition of its activity by specific inhibitor DHEA successfully sensitized TNBC cells, especially the TNBC cells that were derived from lung metastasis, to DOX treatment.

G6PD is an oxidoreductase that plays an important and rate limiting role in PPP through which glucose-6-phosphate is first converted to 6-phosphogluconate, and subsequently to ribose-5-phosphate (Figure 6E). Another important contribution of G6PD is to generate the electron-rich molecules NADPH and GSH, which serve as antioxidants by reducing the oxidative stress produced from DOX and endogenous sources (Figure 6E). Although PPP is a side branch of glycolysis, according to the Warburg effect, cancer cells prefer to use this pathway to metabolize glucose in the cytosol, rather than using oxidative phosphorylation to metabolize glucose-derived pyruvate in mitochondria 35.

As cancer cells can use PPP not only to metabolize glucose but also to produce antioxidants that enable glycolytic cancer cells to combat oxidative stress, more investigations on this pathway and its most important player, G6PD, have been conducted 36,37. For example, it was reported that G6PD protects cells from apoptosis induced by ionizing radiation through maintaining intracellular GSH level 38. In one study using leukemia cell lines, high level of G6PD was found in DOX-resistant cells 39, while in another study using low-metastatic human breast cancer, MCF-7 cell line, the level of G6PD was significantly decreased in DOX-resistant MCF-7 cells 40-42.

In this study, we found that G6PD was upregulated in metastatic TNBC cells that acquired DOX resistance following lung metastasis. More importantly, we demonstrated for the first time that suppression of G6PD abrogated DOX resistance in those metastatic TNBC cells, as well as increased parental TNBC cells' sensitivity to DOX treatment.

In another research work, DOX-resistant human colon cancer cells that displayed increased activity of PPP and G6PD have lower intracellular DOX accumulation in comparison with DOX-sensitive counterpart. The author concluded that high GSH level helps the cells to extrude DOX out of cells 43. Here, we measured DOX concentration in metastatic and parental cells and found there is no significant difference of DOX concentration existed between these two cells lines, thus excluding the possibility of using efflux pumps to mediate DOX resistance acquired after metastasis.

Upon DOX treatment, the metastatic TNBC cells with higher G6PD level exhibited lower apoptotic rate compared to the parental cells. However, when G6PD was suppressed, the apoptotic rate was increased, accompanied with decreased intracellular GSH level. In addition to the antioxidant property of GSH in scavenging hydrogen peroxide, GSH also keeps the important apoptosis mediator, cytochrome c, in its reduced and inactive form after cytochrome c was released from mitochondria 44-46. Therefore, we proposed that stable GSH level maintained by high level of G6PD can enable cancer cells to resist DOX-induced apoptosis, thus conferring metastatic cancer cells with DOX resistance.

Finally, in this study, a non-competitive G6PD inhibitor DHEA 47 successfully reversed intrinsic and metastasis-acquired DOX resistance of TNBC cells. Unfortunately, low water solubility of DHEA as well as limited alternative choices of G6PD inhibitors limit the *in vivo* efficacy study of G6PD inhibitors 48. More potent and specific G6PD inhibitors should be developed, and their clinical values need to be evaluated in the future.

## Abbreviations

Bcl-2: B-cell lymphoma 2
Bcl-xL: B-cell lymphoma-extra-large
DHEA: dehydroepiandrosterone
DOX: doxorubicin
ER: estrogen receptor
FBS: fetal bovine serum
FRET: fluorescence resonance energy transfer
G6PD: glucose-6-phosphate dehydrogenase
GSH: glutathione
HER2: human epidermal growth factor receptor 2
MnSOD: manganese superoxide dismutase
NADPH: nicotinamide adenine dinucleotide phosphate
PPP: pentose phosphate pathway
PR: progesterone receptor
ROS: reactive oxygen species
TNBC: triple negative breast cancer
2D-DIGE: two-dimensional difference gel electrophoresis
